# Barrier-to-Autointegration Factor 1 Protects against a Basal cGAS-STING Response

**DOI:** 10.1128/mBio.00136-20

**Published:** 2020-03-10

**Authors:** Hongming Ma, Wei Qian, Monika Bambouskova, Patrick L. Collins, Sofia I. Porter, Andrea K. Byrum, Rong Zhang, Maxim Artyomov, Eugene M. Oltz, Nima Mosammaparast, Jonathan J. Miner, Michael S. Diamond

**Affiliations:** a Department of Medicine, Washington University School of Medicine, St. Louis, Missouri, USA; b Department of Pathology and Immunology, Washington University School of Medicine, St. Louis, Missouri, USA; c Department of Molecular Microbiology, Washington University School of Medicine, St. Louis, Missouri, USA; d Andrew M. and Jane M. Bursky Center for Human Immunology and Immunotherapy Programs, Washington University School of Medicine, St. Louis, Missouri, USA; e Department of Microbial Infection and Immunity, The Ohio State University, Columbus, Ohio, USA; f Key Laboratory of Medical Molecular Virology (MOE/NHC/CAMS), School of Basic Medical Sciences, Shanghai Medical College, Fudan University, Shanghai, China; UC Berkeley

**Keywords:** interferon-stimulated gene, regulation, innate immunity, CRISPR, DNA virus, RNA virus, antiviral, interferon

## Abstract

Although the pathogen recognition receptor pathways that activate cell-intrinsic antiviral responses are well delineated, less is known about how the host regulates this response to prevent sustained signaling and possible immune-mediated damage. Using a genome-wide CRISPR-Cas9 screening approach to identify host factors that modulate interferon-stimulated gene (ISG) expression, we identified the DNA binding protein Barrier-to-autointegration factor 1 (Banf1), a previously described inhibitor of retrovirus integration, as a modulator of basal cell-intrinsic immunity. Ablation of Banf1 by gene editing resulted in chromatin activation near host defense genes with associated increased expression of ISGs, including *Oas2*, *Rsad2* (viperin), *Ifit1*, and *ISG15*. The phenotype in Banf1-deficient cells occurred through a cGAS-, STING-, and IRF3-dependent signaling axis, was associated with reduced infection of RNA and DNA viruses, and was reversed in Banf1 complemented cells. Confocal microscopy and biochemical studies revealed that a loss of Banf1 expression resulted in higher level of cytosolic double-stranded DNA at baseline. Our study identifies an undescribed role for Banf1 in regulating the levels of cytoplasmic DNA and cGAS-dependent ISG homeostasis and suggests possible therapeutic directions for promoting or inhibiting cell-intrinsic innate immune responses.

## Abstract (precis)

Although the interferon (IFN) signaling pathway is a key host mechanism to restrict infection of a diverse range of viral pathogens, its unrestrained activity either at baseline or in the context of an immune response can result in host cell damage and injury. Here, we used a genome-wide CRISPR-Cas9 screen and identified the DNA binding protein Barrier-to-autointegration factor 1 (Banf1) as a modulator of basal cell-intrinsic immunity. A loss of Banf1 expression resulted in higher level of cytosolic double-stranded DNA at baseline, which triggered IFN-stimulated gene expression via a cGAS-STING-IRF3 axis that did not require type I IFN or STAT1 signaling. Our experiments define a regulatory network in which Banf1 limits basal inflammation by preventing self DNA accumulation in the cytosol.

## INTRODUCTION

Mammalian cells detect and respond to RNA virus infection by recognizing non-self RNA elements through multiple pathogen recognition receptors (PRRs), including the cell surface and endosomal RNA sensors Toll-like receptors 3 and 7 (TLR3 and TLR7), and the cytoplasmic RNA sensors retinoic acid-inducible gene I (RIG-I) and melanoma-differentiation-associated gene 5 (MDA5). Binding of single- and/or double-stranded viral RNA to PRRs results in downstream activation of transcription factors, including interferon (IFN) regulatory factors 3 and 7 (IRF-3 and IRF-7) and NF-κB, and induction of IFN-α and -β. Secretion of IFNs, followed by engagement of the IFN-α/β receptor (IFNAR), in an autocrine and paracrine fashion activates JAK/STAT-dependent signal transduction cascades (1) that induce the expression of hundreds of ISGs, many of which have antiviral activity (2). In addition to using RNA sensors, cells use DNA-sensing machinery to detect DNA viruses or intracellular damage generated early during infection by RNA or DNA viruses (3). As an example, leaked DNA from the mitochondria or nucleus is detected by the DNA sensor cyclic GMP-AMP synthase (cGAS), which triggers the stimulator of IFN genes (STING) and induction of type I IFN and ISGs. At each stage of the immune response, stimulatory and inhibitory signals regulate the magnitude, quality, and character of the response. Positive regulators amplify immune response to clear viral infection, whereas negative regulators dampen inflammatory responses to prevent immune-mediated tissue damage and spontaneous autoimmunity (4).

Several ISGs can temper PRR and type I IFN responses (reviewed in references 4 and 5). As examples, the IFN-induced ring finger protein 125 (RNF125) can target RIG-I, MDA5, and MAVS for ubiquitin-mediated proteasomal degradation (6). Members of the tripartite motif (TRIM) family of E3 ubiquitin ligases inhibit IFN production by targeting of key signaling molecules (e.g., TRIF, TBK1, and IKKβ) or transcription factors (IRF3, IRF7, and NF-κB) for ubiquitin-mediated degradation (7). The suppressor of cytokine signaling (SOCS) family of proteins negatively regulates inflammation by targeting the tyrosine kinase activity of Janus kinases (JAK), which inhibits JAK/STAT signaling pathways and attenuates antiviral responses (8).

We set out to identify novel negative regulators of ISG induction in hopes of identifying targets for inhibition that might allow enhanced viral clearance during acute infections. We performed a genome-wide CRISPR-Cas9 screen in BV2 microglial cells and evaluated for genes that, when edited, resulted in increased cell surface expression of Bst2 (tetherin), a well-described ISG with antiviral activity against multiple enveloped RNA and retroviruses (9, 10). One of our top “hits” was Barrier-to-autointegration factor 1 (Banf1), a small (10 kDa) conserved DNA-binding protein with homeostatic functions in mitosis, nuclear assembly, and the DNA damage response (11, 12) that also can recognize foreign DNA and prevent chromosomal integration (13) or genome replication (14). Banf1 is expressed normally in the inner nuclear membrane but can relocalize to the cytoplasm depending on the stage of cell cycle and age of the cell (15). In addition, Banf1 can bind to exogenous double-stranded DNA in the cytosol after endosomal breakdown to avoid autophagy (16).

In our study, editing of *Banf1* in BV2 mouse microglial cells (Δ*Banf1*) resulted in increased surface expression of Bst2 and higher mRNA levels of several antiviral ISGs, including *Rsad2* and *Oas2*, and this occurred even when type I IFN signaling was blocked. Reciprocally, Δ*Banf1* cells complemented with wild-type *Banf1* demonstrated lower levels of ISGs than control, nonedited cells. These results with control, Δ*Banf1*, and Δ*Banf1*+*Banf1* cells were confirmed by RNA sequencing (RNA-Seq) analysis, and parallel chromatin immunoprecipitation sequencing (ChIP-Seq) studies revealed that Δ*Banf1* cells had active chromatin surrounding several host defense genes. Loss of Banf1 expression was associated with reduced infection of RNA and DNA viruses, and the enhanced expression of ISGs was mediated by a pathway requiring cGAS, STING, and IRF3. Decreased expression of Banf1 also was associated with increased levels of cytosolic double-stranded DNA, which likely triggered recognition by cGAS. Collectively, these results suggest that Banf1, in addition to its established ability to inhibit retrovirus integration and DNA virus replication, regulates levels of endogenous cytoplasmic double-stranded DNA at baseline, which prevents adventitious cGAS-STING activation and cellular ISG responses.

## RESULTS

### A genome-wide CRISPR/Cas9 screen identifies Banf1 as a negative regulator of ISG expression.

**FIG 1:**
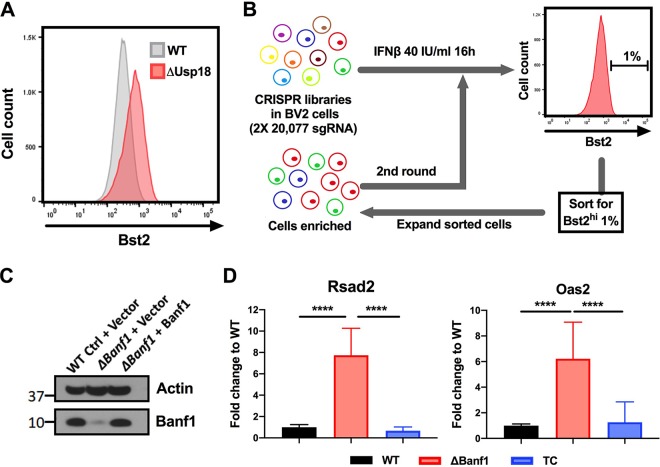
A genome-wide CRISPR screen identified Banf1 as a negative regulator of ISG expression. (A) Bst2, an ISG expressed on the cell surface, was used as a marker for a FACS-based screen for regulators of type I IFN signaling. Cell surface levels of Bst2 were determined after 16 h of IFN-β stimulation in WT or *Usp18*^−/−^ cells. Usp18 is an established negative regulator of type I IFN signaling (17). (B) Flow chart of a FACS-based screen to identify negative regulators of ISG expression. BV2 mouse microglial cells that were transduced with a CRISPR-Cas9 library were treated with a threshold dose of IFN-β and then sorted (top 1%) for high level of Bst2 expression. After expansion, this procedure was repeated once, and then sgRNA sequences were recovered after next-generation sequencing. (C and D) Banf1 was identified as a negative regulator of ISG expression. (C) An immunoblot shows the Banf1 protein levels in different clonal cells (WT and *Banf1* mutant BV2 cells [Δ*Banf1*] complemented with *Banf1* [TC] or empty vector). The data are representative of three experiments. (D) mRNA levels of *Rsad2* and *Oas2*, two representative ISGs, in Δ*Banf1* cells. This phenotype is reversed by the ectopic expression of *Banf1*. The data are normalized to WT control complemented with a control vector and expressed as means ± standard deviations (SD). Three experiments were each performed in triplicate, and the results were assessed using one-way ANOVA with Dunnett’s posttest (****, *P* < 0.0001).

To screen for negative regulators of the IFN pathway, we established a flow cytometry-based strategy with BV2 microglia cells using Bst2 (tetherin), a cell surface protein induced by type I IFNs (Fig. 1A) (9), as an indicator of altered ISG expression (Fig. 1B). Since Usp18 is an established negative regulator of the IFN pathway (17), we generated Δ*Usp18* BV2 cells (see Fig. S1A in the supplemental material) as a positive control to validate the screening approach. When treated with intermediate (10 to 100 IU/ml) doses of IFN-β, Δ*Usp18* BV2 cells differentially showed higher levels of Bst2 on the cell surface than did control cells (Fig. S1B). Based on these data, we used BV2-Cas9 cells with two independent CRISPR libraries, each with greater than 20,000 small guide RNAs (sgRNA) targeting all of the protein-coding genes in the mouse genome (18). The top 1% of cells expressing of the highest levels of Bst2 were sorted by flow cytometry. After expansion in culture, a second round of sorting was performed, and the cells expressing the highest surface levels of Bst2 again were collected. Subsequently, genomic DNA was recovered, deep sequencing of the guide RNAs was performed, and gene enrichment was determined using MAGeCK analysis (19) (Table S1). We manually picked genes (e.g., *Banf1*, *Cmas*, *Dhps*, *Gale*, and *Mgat2*; Fig. S2A) that ranked in the top 150 of both libraries for validation using two independent sgRNA (Fig. S2B). Only one gene, *Banf1*, showed the desired phenotype of elevated levels of ISGs (*Rsad2* [viperin] and *Ifit1*) in the two gene-edited cells compared to control sgRNA cells (Fig. S2C). To rule out possible off-target effects, five additional sgRNA against *Banf1* were tested in bulk cell lines (Fig. S2D); these cells showed that a deficiency of Banf1 expression resulted in enhanced ISG levels at baseline, even in the absence of exogenous IFN-β treatment. To confirm our findings, we generated a clonal Δ*Banf1* BV2 cell line (Fig. 1C and Fig. S2E), which showed elevated levels of ISGs (Rsad2 and Oas2), and performed complementation (*ΔBanf1 *+* Banf1*), which rescued the phenotype (Fig. 1D). Of note, *ΔBanf1* cell line was not a complete null, since small amounts of Banf1 were detected in immunoblots (Fig. 1C); this expression pattern was anticipated as a complete loss of Banf1 is lethal during embryogenesis in invertebrates (20). Notwithstanding this point, the cell viability and metabolic activity of uninfected *ΔBanf1* hypomorphic cells were equivalent to the control or complemented cells (Fig. S2F to G).

10.1128/mBio.00136-20.1 FIG S1 Cell surface Bst2 as a marker for the CRISPR screen. (A) In BV2 cells, *Usp18* was edited using CRISPR/Cas9 as shown in the deep sequencing data. The guide RNA target is highlighted in red. The two alleles with an insertion (middle) or deletion (bottom) are shown. (B) After treatment with low to intermediate (10 to 100 IU/ml) doses of IFN-β, Δ*Usp18* BV2 cells showed higher levels of Bst2 expression on the cell surface than WT cells as judged by flow cytometry. The data are representative of three experiments. Download FIG S1, TIF file, 0.7 MB. Copyright © 2020 Ma et al. 2020 Ma et al. This content is distributed under the terms of the Creative Commons Attribution 4.0 International license.

10.1128/mBio.00136-20.2 FIG S2 Banf1 is a negative regulator of Bst2 expression. (A) The list of top candidate genes for negative regulation of Bst2 expression that were identified in the screen. (B) To validate candidate genes, BV2 cells were edited separately with two independent sgRNA (not from the original library) as shown. (C) *Rsad2* and *Ifit1* were used as markers for ISG induction after editing candidate genes. Cells were treated with 0 or 40 IU/ml IFN-β for 16 h and then subjected to RNA extraction and RT-qPCR. The data are representative of two experiments. (D) To rule out off-target effects, *Banf1* was edited with five additional sgRNA in BV2 cells. The mRNA levels of *Rsad2* and *Ifit1* in these cells were measured with qPCR. The data are representative of two experiments. (E) A clonal Δ*Banf1* BV2 cell was generated and confirmed by deep sequencing. In this clonal line, *Banf1* was not completely edited, with 24.8% WT reads present. (F and G) Loss of Banf1 expression does not diminish cell viability. (F) Cell viability of wild type control (WT), Δ*Banf1*, and Δ*Banf1* complemented (TC) BV2 cells. Equal numbers of cells were plated and cultured for the specified times. Viability was assessed using a luminescent cell viability assay (CellTiter-Glo). (G) Growth of WT, Δ*Banf1*, and TC BV2 cells. Equal numbers of cells were plated and cultured for the specified times. Cell counts were measured by flow cytometry. The data are pooled from three experiments (*n *= 9) and subjected to two-way ANOVA with Tukey’s posttest. Download FIG S2, TIF file, 2.5 MB. Copyright © 2020 Ma et al. 2020 Ma et al. This content is distributed under the terms of the Creative Commons Attribution 4.0 International license.

10.1128/mBio.00136-20.9 TABLE S1 Gene rank of “hits” by MAGeCK analysis. Download Table S1, XLSX file, 0.02 MB. Copyright © 2020 Ma et al. 2020 Ma et al. This content is distributed under the terms of the Creative Commons Attribution 4.0 International license.

### A deficiency in Banf1 results in global ISG expression.

**FIG 2:**
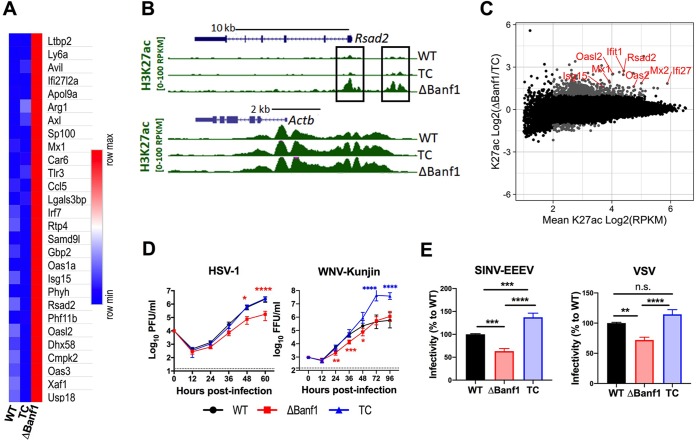
A deficiency of Banf1 results in global ISG upregulation and antiviral effects. (A to D) RNA-Seq and ChIP-seq analyses in control (WT), *ΔBanf1*, and *ΔBanf1*+Banf1 complemented (TC) BV2 cells. (A) Upregulated genes identified with RNA-Seq in Δ*Banf1* cells compared to WT and to TC BV2 cells, with an at least 3× higher expression and a minimum of three RPKM (reads per kb per million). The majority of these genes (26 of 28) are ISGs (http://www.interferome.org/interferome/home.jspx). (B) UCSC Genome Browser snapshots showing gene location and H3K27 acetylation values (RPKM) for *Rsad2* (top) and *Actb* (bottom) loci in control (WT), Δ*Banf1*+*Banf1* complemented (TC), or Δ*Banf1* BV2 cells. The Rsad2 promoter and a nearby putative enhancer are highlighted by rectangular frames. (C) MA (Bland-Altman) plot showing the H3K27 acetylation values from ΔBanf1 or TC cells. Gray dots indicate H3K27 acetylation peaks whose values differ at least 2-fold between groups and are above an average of 4 RPKM. Red labels indicate selected genes proximal (within 10 kb) of indicated H3K27ac peaks. (D) Multistep growth curve of HSV-1 (MOI of 0.05) and WNV-Kunjin virus (MOI of 0.01) in WT, Δ*Banf1*, and TC BV2 cells. The virus titer is expressed as PFU (for HSV-1) or as focus-forming units (FFU; for WNV-Kunjin) per ml. The data are from three experiments with duplicates (for HSV-1) or triplicates (for WNV-Kunjin). Two-way ANOVA with Tukey’s posttest was performed (*, *P* < 0.05; **, *P* < 0.01; ***, *P* < 0.001; ****, *P* < 0.0001). Red asterisks, comparison of ΔBanf1 to WT cells; blue asterisks, comparison of TC to WT cells. Dotted lines indicate limits of detection of the assay. (E) Single-step infection with chimeric SINV-EEEV (MOI of 20, 6 h) and VSV-GFP (MOI of 3, 6.5 h). Infection was measured by flow cytometry. Infectivity is shown as the product of the percentage of infected cells multiplied by the MFI of the positive cells. The data are normalized to values of WT and are shown as means ± SD. Three experiments were each performed in triplicate, and the results were assessed using one-way ANOVA with Dunnett’s posttest (*, *P* < 0.05; **, *P* < 0.01; ***, *P* < 0.001; ****, *P* < 0.0001; n.s., not significant).

Our results suggested a role for Banf1 in regulating basal ISG expression. To evaluate this in greater detail, we paired global transcriptome and epigenetic profiling assays in BV2 cells deficient in Banf1 (*ΔBanf1*), complemented with Banf1 (TC), or control lines (WT). We first conducted RNA-Seq analysis to identify differentially expressed genes. In *ΔBanf1* BV2 cells, highly upregulated genes (cutoff = 3× higher expression than WT cells and >3 reads per kb per million [RPKM]) were mostly ISGs (26 of 28 [21]) (Fig. 2A). Indeed, the gene ontology response pathway to type I IFN (GO 0034340) was highly enriched in *ΔBanf1* BV2 cells (enrichment false discovery rate of 2.79E–11 [22]). We next determined the activation status of genes using histone 3 lysine 27 acetylation (H3K27ac) chromatin immunoprecipitation-sequencing (ChIP-Seq), since this mark correlates with active enhancers and transcription. As an example, the *Rsad2* promoter and a nearby putative enhancer were active in the *ΔBanf1* cells but inactive in WT and TC cells, whereas the *β-actin* gene loci showed no difference in H3K27 acetylation status in any of the three cells (Fig. 2B). To extend these observations, we compared H3K27 acetylation status on a genome-wide level in TC and *ΔBanf1* cells (Fig. 2C and Fig. S3). Most of the regulatory elements with differential H3K27 acetylation were proximal to known ISGs. Indeed, an unbiased analysis using linked differential gene expression with chromatin activation, revealed that the majority of direct targets in the *ΔBanf1* BV2 cells were established ISGs (gene enrichment overlap with interferon alpha response *P* = 5e–17 [hypergenometric test]).

10.1128/mBio.00136-20.3 FIG S3 Differential expression of genes proximal to histone acetylation in *ΔBanf1* and complemented cells. Relative expression of genes proximal to H3K27 acetylation peaks in Δ*Banf1* (KO) or TC cells. Genes whose RPKM values change by at least 4-fold and are within 10 kb of a differentially regulated H3K27 acetylation peak between the two cell types are shown. Download FIG S3, TIF file, 0.5 MB. Copyright © 2020 Ma et al. 2020 Ma et al. This content is distributed under the terms of the Creative Commons Attribution 4.0 International license.

### A deficiency in Banf1 is associated with reduced viral infection.

Given the higher basal level of ISGs in *ΔBanf1* cells, we hypothesized they might contribute to resistance to virus infections. To evaluate this idea, we assessed infection of positive-sense RNA viruses including a flavivirus (West Nile virus [WNV]-Kunjin virus) and an alphavirus (chimeric Eastern equine encephalitis virus (SINV-EEEV), a negative-sense RNA virus (vesicular stomatitis virus [VSV]), and a DNA virus (herpes simplex virus 1 [HSV-1]) in *ΔBanf1* BV2 cells; these viruses were selected because they replicated efficiently in parental BV2 cells. Multistep growth curve analysis showed that a deficiency of Banf1 was associated with less infection by HSV-1 and WNV-Kunjin compared to control cells (Fig. 2D). To evaluate further the effect of Banf1 expression on the basal or rapidly induced ISG response, we analyzed viral replication under inoculation conditions with high and low multiplicities of infection with SINV-EEEV and VSV. These experiments showed that *ΔBanf1* BV2 cells were more resistant than control cells (Fig. 2E and Fig. S4). Reciprocally, *ΔBanf1 *+* Banf1* complemented BV2 cells, which had lower levels of ISG mRNA at baseline (Fig. 2A and C), showed greater infection with WNV-Kunjin and SINV-EEEV (Fig. 2D and E and Fig. S4).

10.1128/mBio.00136-20.4 FIG S4 A deficiency of Banf1 results greater viral infection. Infection with chimeric SINV-EEEV-GFP (MOI of 0.001, 30 h) and VSV-GFP (MOI of 0.001, 18 h). Infection was measured by flow cytometry. Infectivity is shown as the product of the percentage of infected cells multiplied by the median of the fluorescence intensity of the positive cells. The data are normalized to values of WT and shown as means ± SD. Three experiments were each performed in quadruplicate or quintuplicate, and the results were assessed using one-way ANOVA with Dunnett’s posttest (****, *P* < 0.0001). Download FIG S4, TIF file, 0.3 MB. Copyright © 2020 Ma et al. 2020 Ma et al. This content is distributed under the terms of the Creative Commons Attribution 4.0 International license.

### Basal ISG expression in *ΔBanf1* cells is independent of type I IFN signaling.

**FIG 3:**
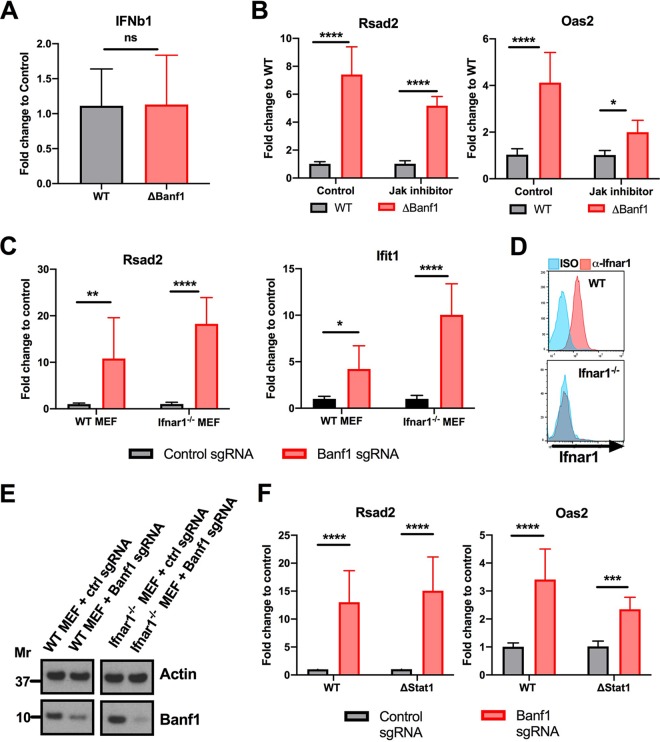
ISGs are upregulated in ΔBanf1 cells independently of type I IFN signaling. (A) *IFNβ1* gene expression in WT and ΔBanf1 BV2 cells. Three experiments were each performed in triplicate, and the results were assessed using an unpaired Student *t* test on pooled data (n.s., not significant). (B) Inhibition of JAK signaling activity with 10 μM ruxolitinib does not affect ISG (*Rsad2*, left; *Oas2*, right) upregulation phenotype in *ΔBanf1* BV2 cells. (C) WT and *Ifnar1*^−/−^ MEFs that were edited via CRISPR-Cas9 with a control or Banf1 sgRNA and evaluated for ISG (*Rsad2* and *Ifit1*) expression at baseline by qRT-PCR. (D) The Ifnar1 deficiency was confirmed by flow cytometry. (E) Banf1 expression was reduced after gene editing as shown by immunoblotting. (F) The *Stat1* gene was edited in BV2 cells. WT cells and a clonal Δ*Stat1* BV2 cells (Fig. S5) were then edited with a control or Banf1 sgRNA and evaluated for ISG (*Rsad2* and *Oas2*) expression by qRT-PCR. (B, C, and F) Data are means ± SD. Three experiments were each performed in triplicate, and the results were assessed using two-way ANOVA with Sidak’s posttest on pooled data (*, *P* < 0.05; **, *P* < 0.01; ***, *P* < 0.001; ****, *P* < 0.0001).

Our initial experiments showed that the induction of ISGs associated with decreased Banf1 expression did not require exogenous IFN-β administration (Fig. S2C-D). We also found no upregulation of IFN-β mRNA in *ΔBanf1* cells (Fig. 3A). Since ISG induction can happen in a type I IFN-independent manner (e.g., via IRF3 activation [23]), we first assessed whether the IFN signaling pathway was required for the ISG phenotype in *ΔBanf1* cells. Administration of an inhibitor of Jak activity did not fully rescue the ISG upregulation phenotype (Fig. 3B). We next edited Banf1 expression in wild-type (WT) and *Ifnar1*^−/−^ murine embryonic fibroblasts (MEFs). As expected, *ΔBanf1* WT MEFs had higher levels of ISGs (e.g., *Rsad2* and *Ifit1*) than control gene-edited WT MEFs. Remarkably, *ΔBanf1 Ifnar1*^−/−^ MEFs had a similar phenotype as *ΔBanf1* WT MEFs (Fig. 3C to E). We confirmed these findings in *ΔStat1* gene-edited BV2 cells (Fig. 3F and Fig. S5A and B). Thus, in BV2 cells and fibroblasts, the ISG induction associated with loss of Banf1 expression occurred independently of type I IFN signaling.

10.1128/mBio.00136-20.5 FIG S5 Gene editing of Banf1 in Δ*Stat1* BV2. (A) Stat1 was edited using CRISPR/Cas9 as shown in deep sequencing data. The guide RNA target is highlighted in red. The three alleles with indel causing frame shift are shown. (B) *Banf1* was edited in WT and *ΔStat1* BV2 cells using CRISPR/Cas9-based targeting, and Banf1 protein expression is shown by immunoblotting. Download FIG S5, TIF file, 0.6 MB. Copyright © 2020 Ma et al. 2020 Ma et al. This content is distributed under the terms of the Creative Commons Attribution 4.0 International license.

### ISG expression in *ΔBanf1* cells occurs via a cGAS/STING/IRF3 pathway.

**FIG 4:**
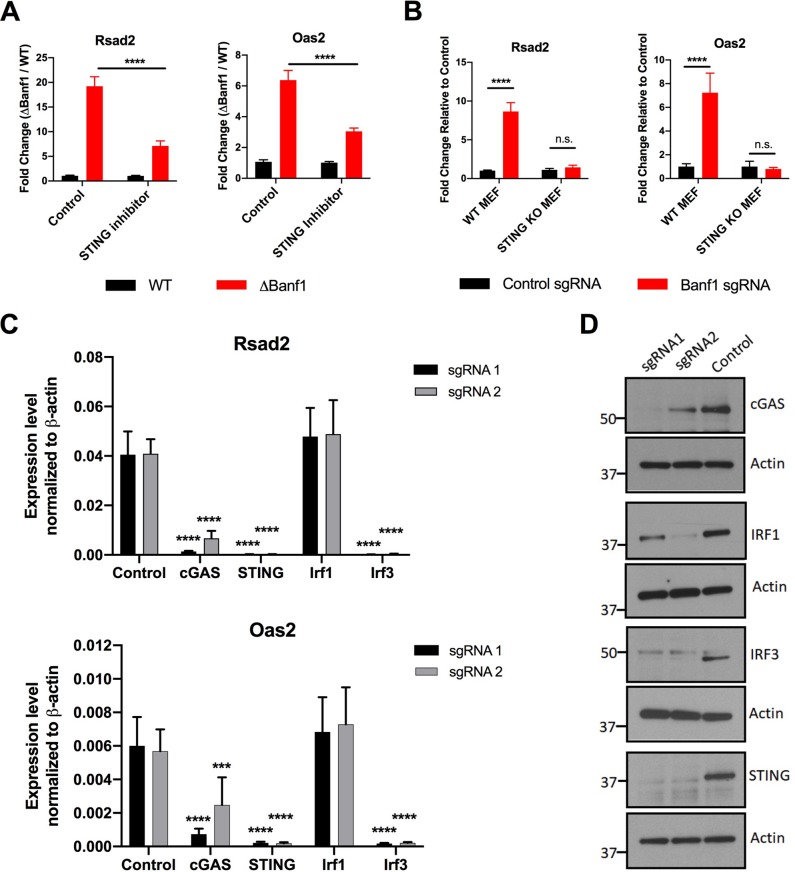
ISG upregulation in *ΔBanf1* cells occurs via a cGAS-STING-IRF3 pathway. (A) In *ΔBanf1* BV2 cells, the ISG induction phenotype is rescued partially by treatment with NO_2_-FA (10 μM for 15 min), an inhibitor of STING. Three experiments were each performed in triplicate, and the results were assessed using two-way ANOVA (****, *P* < 0.0001). (B) *Banf1* was edited using CRISPR-Cas9 in STING^−/−^ and WT MEFs. Expression of *Rsad2* and *Oas2* mRNA is shown. Three experiments were each performed in triplicate, and the results were assessed using two-way ANOVA (****, *P* < 0.0001). (C) The indicated DNA sensors and signaling molecules were edited using CRISPR-Cas9 in *ΔBanf1* BV2 cells. Two guide RNAs (sgRNA) were used for each gene. The expression levels of *Rsad2* (top) and *Oas2* (bottom) mRNA are shown and were normalized to β-actin. Three experiments were each performed in triplicate, and the results were assessed using one-way ANOVA with Dunnett’s posttest (***, *P* < 0.001; ****, *P* < 0.0001). (D) Immunoblotting of cGAS, STING, Irf1, Irf3, and β-actin in gene-edited BV2 cells. The cell sample for Irf1 was treated with IFN-γ (50 ng/ml for 4 h) before harvesting and lysis. The data are representative of two experiments.

Prior studies have suggested that Banf1 might regulate DNA damage responses (11). We hypothesized that a loss of Banf1 expression might trigger sensing of self-DNA and innate immune signaling. To evaluate this hypothesis, we first tested whether a pharmacological inhibitor of STING (NO_2_-FA [24]) could rescue the ISG phenotype in *ΔBanf1* BV2 cells. At concentrations that do not cause cellular cytotoxicity (Fig. S6A), treatment with the STING inhibitor partially reduced ISG expression at baseline in *ΔBanf1* but not wild-type control cells (Fig. 4A). To corroborate these results, we edited Banf1 expression in WT and STING KO (25) MEFs (Fig. S6B). In wild-type MEFs, loss of Banf1 expression resulted in upregulation of several ISGs. In contrast, in STING KO cells, editing of Banf1 had no impact on ISG expression (Fig. 4B). Given that the Banf1-dependent ISG phenotype was mediated through STING, we next defined the pathway by editing a more comprehensive set of DNA sensors (e.g., Aim2, cGAS, Ddx41, Ifi204, Pqbp1, Pyhin1, and Zbp1) and signaling molecules (e.g., STING, Irf1, Irf3, and Irf8) in *ΔBanf1* BV2 cells. Among the target genes tested, only editing of cGAS, STING, and Irf3 resulted in loss of ISG expression in *ΔBanf1* BV2 cells (Fig. 4C and D and Fig. S7).

10.1128/mBio.00136-20.6 FIG S6 Cytotoxicity assay of STING inhibitor and expression of Banf1 in STING-deficient cells. (A) Cytotoxicity of the STING inhibitor (NO_2_-FA) was evaluated with luminescent cell viability assay (CellTiter-Glo). Cells were treated with vehicle, control lipid, or STING inhibitor (NO_2_-FA) for 15 min, washed with fresh DMEM media and cultured for 10 h and then subjected to the cell viability assay. The concentration of 10 μM of NO_2_-FA used in the study showed no significant cell viability reduction. As a positive control, the 100 μM concentration caused a decrease in cell viability. Data from two experiments were pooled and analyzed using two-way ANOVA and Sidak’s posttest. (*, *P* < 0.05; **, *P* < 0.01; n.s., not significant). (B) *Banf1* was edited in WT and STING KO MEFs (25) using CRISPR/Cas9-based targeting, and Banf1 protein expression is shown by immunoblotting. Download FIG S6, TIF file, 0.6 MB. Copyright © 2020 Ma et al. 2020 Ma et al. This content is distributed under the terms of the Creative Commons Attribution 4.0 International license.

10.1128/mBio.00136-20.7 FIG S7 ISG upregulation in *ΔBanf1* cells occurs independently of selected DNA sensors and signaling pathways. (A) Several additional DNA sensors and signaling molecules were edited using CRISPR-Cas9 in *ΔBanf1* BV2 cells. Two guide RNAs (sgRNA) were used for each gene. Expression levels of *Rsad2* (*top*) and *Oas2* (*bottom*) mRNA are shown and normalized to β-actin. Three experiments were each performed in triplicate, and the results were assessed using one-way ANOVA with Dunnett’s posttest (*, *P* < 0.05; **, *P* < 0.01). (B) Immunoblotting of Aim2, Ddx41, Pqbp1, and β-actin in gene edited BV2 cells. Note that specific immunoblotting reagents for Pyhin1 were not available. The data are representative of two experiments. Download FIG S7, TIF file, 0.9 MB. Copyright © 2020 Ma et al. 2020 Ma et al. This content is distributed under the terms of the Creative Commons Attribution 4.0 International license.

### A deficiency in Banf1 results in accumulation of double-stranded DNA in the cytosol.

A recently published CRISPR/Cas9 screen showed that STAG2 regulated the cGAS pathway by preventing a DNA damage response (26); accordingly, a loss of STAG2 expression resulted in increased levels of cytoplasmic DNA and induction of type I IFNs and ISGs. Given the apparent similarities, we evaluated whether a deficiency of Banf1 resulted in activation of the DNA damage response by measuring phosphorylation of the histone variant H2AX (γH2AX). However, we did not observe higher levels of γH2AX in Banf1 deficient BV2 cells and MEFs relative to control (WT) or Banf1 complemented cells, as judged by Western blotting (Fig. S8A and B) or immunofluorescence microscopy (Fig. S8C to E). Moreover, a loss of expression of Banf1 also was not associated with formation of micronuclei in these cells (Fig. S8F). Thus, Banf1 and STAG2, despite their similar effects on the cGAS-STING-IRF3 signaling pathway, likely act through different mechanisms. We also inhibited the replication of endogenous retroviruses with antiretroviral nucleoside inhibitors but observed no effect on basal ISG expression in cells lacking Banf1 (Fig. S8G).

10.1128/mBio.00136-20.8 FIG S8 A deficiency of Banf1 does not lead to substantial DNA damage in BV2 cells and MEFs. (A) An immunoblot probing the DNA damage marker phospho-histone H2AX (γH2AX) in WT, ΔBanf1 and TC BV2 cells. Actin and H2AX were included as loading controls. Images are representative of three experiments. (B) *Banf1* was edited in MEFs with CRISPR-Cas9 (Δ*Banf1*) and complemented with *Banf1* (TC). Immunoblotting shows the protein level of Banf1 in WT, Δ*Banf1* and TC MEFs, as well as the corresponding γH2AX levels. (C) Epifluorescence microscopy shows γH2AX foci (anti-γH2AX antibody, red) in WT and Δ*Banf1* MEFs. DNA was stained with Hoechst (blue). (D to F) γH2AX foci and micronuclei were quantified with cellSens Dimension software. At least 100 cells were analyzed in triplicate and a Student two-tailed *t*-test was used to determine statistical significance. (D) Percentage of cells with more than 10 γH2AX foci in WT and *ΔBanf1* MEFs. Three experiments, no statistically significant difference. (E) Signal intensity of γH2AX foci in WT and *ΔBanf1* MEFs. Three experiments, no statistically significant difference. (F) Percentages of cells with micronuclei in WT and *ΔBanf1* MEFs. Three experiments were performed; there was no statistically significant difference. (G) WT and Δ*Banf1* BV2 cells were treated with 10 μM of the nucleoside reverse transcriptase inhibitor lamivudine (3TC) for 2 weeks. ISG (*Rasd2* and *Oas2*) expression was evaluated by qRT-PCR. The data are pooled from three experiments (one-way ANOVA with Dunnett’s posttest; ****, *P* < 0.0001; n.s., not significant). Download FIG S8, TIF file, 1.2 MB. Copyright © 2020 Ma et al. 2020 Ma et al. This content is distributed under the terms of the Creative Commons Attribution 4.0 International license.

**FIG 5:**
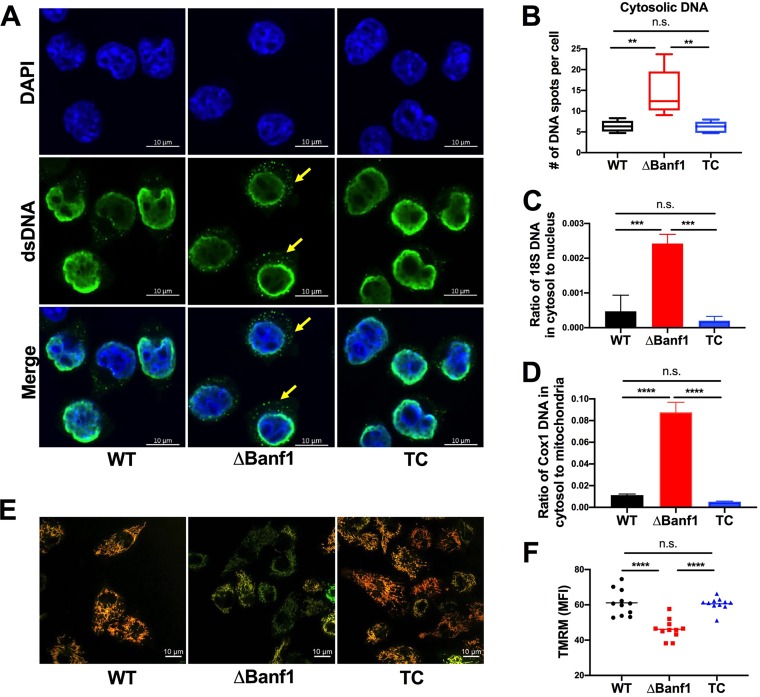
Higher levels of double-stranded DNA in the cytosol of Banf1-deficient cells. (A) Cytoplasmic DNA was evaluated in wild-type (WT), Δ*Banf1*, and *Banf1* complemented (TC) Δ*Banf1* BV2 cells using an anti-double-stranded DNA antibody. Confocal microscopy shows cytoplasmic DNA spots (anti-double-stranded DNA, green) and nuclei (DAPI, blue). Scale bar, 10 μm. Five experiments were conducted, with 15 to 20 fields viewed each. (B) Cytoplasmic DNA spots were quantified with Velocity imaging software for 300 to 1,000 cells of each experiment. The number of double-stranded DNA spots per cell was compared using a one-way ANOVA with Dunnett’s posttest (**, *P* < 0.01; n.s., not significant). (C) The abundance of a nuclear *18S* gene in the cytosol and nucleus was measured with qPCR in WT, *ΔBanf1*, and TC BV2 cells (three experiments; one-way ANOVA with Dunnett’s posttest; ***, *P* < 0.001; n.s., not significant). (D) The mitochondrial gene *Cox1* also shows higher levels in cytosol of *ΔBanf1* BV2 cells than WT and TC cells (three experiments; one-way ANOVA with Dunnett’s posttest; ****, *P* < 0.0001; n.s., not significant). (E and F) A loss of Banf1 expression results in decreased potential of the mitochondrial membrane. (E) Mitochondria were stained with MitoTracker Green (green, independent of mitochondria membrane potential) and tetramethylrhodamine methyl ester (TMRM, red, requires intact mitochondrial potential). In *ΔBanf1* BV2 cells, less TMRM staining is seen. The images are representative of three experiments. (F) The level of TMRM staining was quantified with flow cytometry. Three experiments were each performed, and the results were assessed using one-way ANOVA with Dunnett’s posttest (***, *P* < 0.001; n.s., not significant).

Because of the effects on cGAS activation, we next assessed by confocal microscopy whether a loss of Banf1 expression resulted in basal accumulation of DNA in the cytosol. Indeed, we observed substantially higher levels of double-stranded DNA puncta in the cytosol of *ΔBanf1* BV2 cells than WT or Banf1-complemented cells (Fig. 5A and B). To corroborate these findings, we performed cell fractionation and PCR experiments to quantify the relative abundance of nuclear (*18S*) and mitochondrial (*Cox1*) gene DNA in the cytosol of the different BV2 cells. The cytosolic levels of both the *18S* nuclear and *Cox1* mitochondrial genes were higher in *ΔBanf1* BV2 cells than in WT and Banf1-complemented cells (Fig. 5C and D). To assess the effect of a loss of Banf1 expression on mitochondrial morphology, we performed additional staining. Although no substantive change in mitochondrial morphology was observed in *Banf1* BV2 cells, a deficiency of Banf1 was associated with decreases in mitochondrial membrane potential (Fig. 5E and F), which is an indicator of mitochondrial permeability (27).

## DISCUSSION

Using a CRISPR-Cas9 genome-wide screen that was designed to identify negative regulators of the IFN signaling pathway and ISG expression, we identified Banf1 as a gene that inhibited cell surface expression of Bst2, a well-characterized ISG. However, rather than modulate type I IFN signaling directly, editing of Banf1 resulted in greater basal expression of multiple ISGs, and this effect occurred through a cGAS-STING-IRF3 pathway. Unexpectedly, our screen failed to identify any established negative regulators (e.g., SOCS genes or others [5]) of the IFNAR signaling pathway. Although further analysis is warranted, the dose or timing of exposure of IFN-β used may have desensitized an IFN signaling response, which could have precluded the identification of negative regulators of the pathway.

When Banf1 expression was reduced, we observed global changes in transcription of ISGs and alteration of the epigenetic state with histone acetylation of the ISG promoters. Mechanistic studies revealed that Banf1 prevents accumulation of self, double-stranded DNA in cytosol, which minimizes recognition by cGAS and activation of the STING pathway. A loss of expression of Banf1 expression in BV2 cells or MEFs, however, was not associated with diminished cell viability or rates of proliferation or induction of canonical DNA damage responses. Instead, double-stranded DNA accumulated in the cytoplasm of *ΔBanf1* cells from both nuclear and mitochondrial sources. The higher levels of double-stranded DNA resulted in basal ISG expression, which reduced infection of several RNA and DNA viruses. Reciprocally, ectopic expression of Banf1 resulted in lower ISG levels at baseline and greater WNV-Kunjin and SINV-EEEV infection. Together, these findings suggest that Banf1 expression levels under homeostatic conditions may regulate amounts of double-stranded DNA in the cytoplasm and help to set the basal ISG tone that primes antiviral defenses. Further studies are required to assess whether immune and nonimmune cells and quiescent and dividing cells differentially express Banf1 and ISGs. It is plausible that some cells might regulate basal expression of ISGs in a cGAS-dependent manner (28) by modulating expression of Banf1. Notwithstanding our findings showing that Banf1 expression attenuates basal expression of ISGs, in other contexts Banf1 can have direct antiviral effects. With retroviruses, Banf1 acts as a barrier to chromosomal integration (13) and, for poxviruses, it acts to inhibit replication of exogenous viral DNA in the cytoplasm (29).

Our initial analysis suggested that Banf1 might act analogously to other recently described genes that modulate cGAS activation, type I IFN production, and ISG expression, including Trex1 (30), ATM (31), LATS1/2 (32), and STAG2 (26), as well as the genes causing Fanconi anemia (33) and Hutchinson-Gilford progeria syndrome (34). However, these studies suggested that the DNA damage responses or activation of retroviral or transposon elements from the chromosome are the source of DNA that activates the intracellular sensing and innate immune signaling pathways. Unexpectedly, in the Banf1-deficient cells we tested, we did not detect activation of the DNA damage response as judged by an absence of expression of the marker γH2AX or evidence of staining in cytoplasm. In comparison, some individuals with progeroid syndrome encode a homozygous mutation in Banf1 that impairs protein stability and is associated with fibroblasts having nuclear lamina abnormalities (35). It remains possible that nuclear chromatin fragments in the cytoplasm exist in Banf1-deficient cells; however, these are too small to activate canonical pH2A.X signaling, but they are large enough to be sensed by cGAS.

In separate studies, we inhibited the replication of endogenous retroviruses with antiretroviral nucleoside inhibitors but observed no effect on basal ISG expression in cells lacking Banf1. In both BV2 microglial cells and MEFs, the enhanced ISG expression observed in the setting of reduced expression of Banf1 occurred independently of type I IFN signaling or Stat1 expression. However, Banf1-modulated ISG expression was dependent on IRF3, which can regulate a set of ISGs directly, independently of IFN expression or signaling (23). Future studies in additional cell types are warranted to define why a loss of Banf1 expression and activation of a cytosolic recognition occurs through an IRF3-dependent yet IFN-independent pathway.

In cells from premature aging syndromes, damaged nuclear DNA leaks to the cytosol, accumulates when the machinery to remove or degrade this DNA malfunctions, and induces an inflammatory cytokine response via the STING pathway (36, 37). Banf1 is a component of the nuclear lamina and can bind double-stranded DNA as a highly ordered nucleoprotein complex in a sequence-independent manner (38). Indeed, Banf1 acts as a bridge between chromosomal DNA and nuclear membrane proteins and has a critical function in nuclear reassembly during mitosis (11, 39). Consistent with these data, although we reduced Banf1 expression using multiple sgRNA and gene editing in both BV2 cells and MEFs, we never obtained a complete null, as determined by gene sequencing and immunoblotting. Banf1 protein also was observed to accumulate at sites of nuclear envelope rupture in the context of cancer cell migration and deformation through tight interstitial spaces (40). These studies link Banf1 to control or protection against accumulation of double-stranded DNA in the cytoplasm presumably from nuclear sources. Indeed, we observed accumulation of nuclear DNA in the cytosol in cells deficient in the expression of Banf1, which could accumulate over multiple rounds of cell division even without marked effects on cell division or viability. Beyond its role in limiting accumulation of double-stranded DNA from nuclear sources, our experiments suggest that Banf1 also may directly or indirectly affect mitochondrial integrity or function. Our cell fractionation studies showed an enrichment of mitochondrial DNA in the cytosol in Banf1-deficient cells, and this was associated with altered mitochondrial potential but not morphology. Even moderate mitochondrial stress in Banf1-deficient cells might lead to the release of damage-associated molecular patterns that activate innate immunity via cGAS and STING. What remains uncertain is the linkage between Banf1 and mitochondrial integrity, and whether Banf1 has additional functions in regulating mitophagy, which limits activation of cGAS and STING by minimizing levels of cytosolic mitochondrial DNA (41). Another unresolved question is where cGAS localizes in Banf1-deficient cells to trigger double-stranded DNA binding and ISG induction. Although we attempted to perform immunofluorescence microscopy with commercial anti-mouse cGAS antibodies, these results were difficult to interpret because of background staining in cGAS^−/−^ cells. In the field, consensus is lacking as to where cGAS recognizes self and foreign DNA. In addition to its described localization in the cytosol (42), cGAS can be tethered to the nucleus (43) or localized to the plasma membrane via the actions of an N-terminal phosphoinositide-binding domain (44).

Overall, our studies add to an emerging literature that defines how different families of proteins regulate accumulation of host DNA in the cytoplasm. This regulatory network is critical for cellular homeostasis to enable a rapid innate immune response to pathogens but also limit adventitious inflammation under settings (e.g., cell division, stress, and deformation) when self DNA may accumulate in the cytosol.

## MATERIALS AND METHODS

### Cells, viruses and reagents.

BV2 microglial cells (a gift from Stephanie Karst, University of Florida), C57BL/6 MEFs, and BHK-21, Vero, and HEK 293T cells (ATCC) were cultured in Dulbecco modified Eagle medium (DMEM) with 10% fetal bovine serum (FBS), 1% HEPES, and 1% penicillin-streptomycin unless otherwise indicated. For BV2 cells and MEFs, 2.5 μg/ml of puromycin (InvivoGen), 4 μg/ml blasticidin (InvivoGen), and 200 μg/ml hygromycin (InvivoGen) were added for plasmid selection depending on the application.

The following viruses were used: chimeric SINV-EEEV-GFP (45), VSV-GFP (Indiana) (46), HSV-1 (strain 17) (47), and WNV-Kunjin (48). Because EEEV is a BSL-3 select agent pathogen, we used the chimeric SINV-EEEV BSL-2 pathogen that incorporates the nonstructural genes and RNA replication control elements of a Sindbis virus (SINV; strain TR339) with the structural genes (C-E3-E2-6K-E1) of an EEEV isolate (strain FL93-939); the green fluorescent protein (GFP) reporter gene was added to an additional subgenomic promoter. SINV-EEEV-GFP was propagated in BHK-21 cells, and titers were determined by a focus forming assay (FFA). WNV-Kunjin was propagated in Vero cells and titrated by FFA, and VSV-GFP and HSV-1 were propagated in Vero cells and titrated by plaque assays. The Jak1/2 inhibitor ruxolitinib (INCB018424) was purchased from Selleckchem (catalog no. S1378). Cells were treated at 10 μM ruxolitinib or vehicle for 12 h. The STING signaling inhibitor NO2-FA was obtained (Cayman Chemicals), together with control lipids. Cells were treated with 10 μM NO2-FA for 15 min and washed with fresh media, and samples were harvested 10 h later to minimize possible cytotoxic effects. The nucleoside reverse transcriptase inhibitor (lamivudine [3TC]) used in this study was USP grade and obtained commercially (Aurobindo Pharma). Cells were treated with 10 μM 3TC for 2 weeks and harvested for quantitative reverse transcription-PCR (qRT-PCR).

Cell viability and cytotoxicity assays were conducted using the luminescent cell viability assay (CellTiter-Glo; Promega) according to the manufacturer’s instructions. Luminescence was analyzed on a reader (BioTek Synergy H1) at room temperature with a 0.5-s integration time per well. Cell counting was conducted by flow cytometry (MACSQuant 10; Miltenyi Biotec).

### Gene editing with CRISPR/Cas9.

Oligonucleotides for sgRNA (for the sequences, see Table S2A) were synthesized commercially (Integrated DNA Technology), cloned into the plasmid lentiCRISPR v.2 (Addgene 52961), and packaged by cotransfection with two helper plasmids (psPAX2 [Addgene, catalog no. 12260] and pMD2.G [Addgene, catalog no. 12259]) into HEK-293T cells with Lipofectamine 3000 (Invitrogen) according to the manufacturer’s instructions. The sgRNAs selected were the top two optimized guides from a designed library for maximum gene-editing activity and minimum off-target effects (49). Cells were transduced with lentiviruses expressing individual sgRNA and selected with puromycin for 7 days. An irrelevant sgRNA (GAAGTTCGAGGGCGACACCC) with no target in the mouse genome also was transduced as a negative control. For some experiments, cells were isolated by limiting-dilution single-cell cloning. Gene editing at the target site was evaluated by next generation sequencing on an Illunima HiSeq 2500 platform (Genome Technology Access Center, Washington University) and/or Western blotting.

10.1128/mBio.00136-20.10 TABLE S2 sgRNA and qPCR primer sequences. (A) sgRNA sequences; (B) qPCR primers. Download Table S2, DOCX file, 0.1 MB. Copyright © 2020 Ma et al. 2020 Ma et al. This content is distributed under the terms of the Creative Commons Attribution 4.0 International license.

### Flow cytometry.

For staining of cell surface antigen, cells were detached with Cellstripper (Corning) and resuspended in PBS with 0.5% FBS and 1 mM EDTA (FACS [fluorescence-activated cell sorting] buffer). Bst2 was stained with phycoerythrin (PE)-labeled anti-Bst2 (BioLegend, clone 129C1; 1:500 in FACS buffer) and anti-mouse CD16/CD32 (eBioscience; 1:250 in FACS buffer) on ice for 45 min. Ifnar1 was stained with 1 μg/ml of anti-mouse Ifnar1 (clone MAR1-5A3; BioXcell) at room temperature for 45 min, followed by incubation with a goat anti-mouse IgG (H+L) Alexa Fluor 647 (Thermo Fisher, catalog no. A21236; 1:1,000 in FACS buffer) at room temperature for 30 min. For cells infected with GFP-labeled viruses (SINV-EEEV-GFP and VSV-GFP), cells were detached with trypsin and fixed with 1% paraformaldehyde (PFA) at room temperature for 15 min. Flow cytometry was performed on a MACSQuant Analyzer 10 (Miltenyi Biotec), and data were analyzed using FlowJo 10.6.1.

### CRISPR-Cas9 genome-wide screen.

The mouse Asiago sgRNA CRISPR library in BV2 cells has been published (18) and was a gift (Herbert Virgin, Washington University). The library contains four independent sublibraries, each containing a unique sgRNA targeting each of 20,077 mouse genes (18, 49). We used two of the sublibraries (1 and 5) for the screen. For each sublibrary, 2 × 10^7^ cells containing sgRNAs were seeded into two T-175 tissue culture flasks and cultured for 16 h. Cells were treated with 40 IU/ml mouse IFN-β (PBL Assay Science) for 16 h and detached with Cellstripper (Corning). Cells were incubated with PE-labeled anti-Bst2 (BioLegend, clone 129C1) and anti-mouse CD16/CD32 (eBioscience) on ice for 45 min. The cells were sorted for the population with highest (top 1%) surface expression of Bst2 using a MoFlo high-speed cell sorter (Beckman Coulter, Siteman Flow Cytometry Core, Washington University) and expanded in DMEM supplemented with 10% FBS. When enough cells accumulated (2 × 10^7^), a second round of sorting was conducted. Subsequently, genomic DNA was extracted from the control cells (2 × 10^7^) and sorted cells (1 × 10^7^), respectively. The sgRNA enriched was amplified and subjected to next generation sequencing using an Illumina HiSeq 2500 platform (Genome Technology Access Center, Washington University). The sgRNA sequences against specific genes were determined after removal of the tag sequences using the FASTX-Toolkit (http://hannonlab.cshl.edu/fastx_toolkit/) and cutadapt 1.8.1. sgRNA sequences were analyzed using a published computational tool (MAGeCK) (19) (see Table S1).

### Hit validation.

Genes with the highest rankings in both library screens were validated using two independent sgRNAs (Fig. S2). The sgRNAs were cloned into the plasmid lentiCRISPR v.2; lentiviruses were generated and BV2 cells were transduced as described above. An irrelevant sgRNA (GAAGTTCGAGGGCGACACCC) with no target in the mouse genome also was transduced as a control.

For complementation of *Banf1* expression, the full-length mouse *Banf1* open reading frame corresponding to the transcript (NM_001038231.2) was synthesized (Integrated DNA Technologies) and cloned into the lentivirus vector pLV-EF1α-IRES-BLAST (Addgene 85133) between BamHI and MluI restriction enzyme sties. The *Banf1* sgRNA target sequence and the PAM sequence were mutated synonymously (TGACGTCCTGAGCAAGAGGC to TGACGTCCTGAGTAAAAGAC; TGG to TCG) to avoid recutting of the reintroduced gene. Lentiviruses were generated as described above. Cells transduced with lentiviruses were selected with 4 μg/ml blasticidin (InvivoGen) for 7 days. An empty vector was transduced as a negative control. The ectopic expression of Banf1 was confirmed with Western blotting.

### Viral infections.

BV2 cells were inoculated with SINV-EEEV-GFP (multiplicity of infection [MOI] of 20) for 6 h or VSV-GFP (MOI of 3) for 6.5 h. Low-MOI infections also were conducted using an MOI of 0.001 for 30 h for SINV-EEEV-GFP and an MOI of 0.001 for 18 h for VSV-GFP. Cells were harvested using trypsin digestion and fixed in 1% PFA for 15 min at room temperature. Cells then were subjected to flow cytometric analysis (MACSQuant Analyzer 10; Miltenyi Biotec) using FlowJo software (Tree Star). Virus infection was defined as a product of the percentage of GFP-positive cells multiplied by the mean fluorescence intensity (MFI) of the positive cells. Growth curves of HSV-1 and WNV-Kunjin was conducted by inoculating BV2 cells at MOIs of 0.05 and 0.01, respectively. Supernatants were collected at specified time points, and titers were determined with a plaque-forming assay (HSV-1) or an FFA (WNV-Kunjin).

### Western blotting.

Cell lysates were prepared using radioimmunoprecipitation assay (RIPA) buffer (Thermo Fisher) supplemented with a cocktail of protease and phosphatase inhibitors (Thermo Fisher) on ice. Protein concentration was determined with a BCA protein assay (Pierce, catalog no. 23227). Gel electrophoresis was performed on 4 to 12% Bis-Tris NuPAGE gel (Invitrogen) and transferred onto polyvinylidene difluoride membranes according to the manufacturer’s instructions (iBLOT2; Life Technologies). Membranes were blotted with the following primary antibodies: Banf1 (BAF antibody A-11 [Santa Cruz, sc-166324]; 1:500), Irf3 (CST 4302; 1:1,000), cGAS (CST 31659; 1:1,000), Irf1 (CST 8478; 1:1,000), Aim2 (CST 63660; 1:1,000), Ddx41 (CST 15076; 1:1,000), Pqbp1 (Proteintech 16264-1-AP; 1:500), γH2AX (phospho S139) (Abcam, ab26350 [1:5,000] or CST 9718 [1:1,000]), H2AX (CST 2595; 1:1,000), actin (CST 3700; 1:3,000) and STING (CST 13647; 1:1,000). Horseradish peroxidase-conjugated goat anti-rabbit (Pierce, 31460; 1:5,000) or anti-mouse (Sigma, A8924; 1:5,000) IgG was used as a secondary antibody. Protein bands were visualized with chemiluminescence substrate (Thermo Fisher, 34577) and autoradiograph film (MIDSCI).

### Gene expression analysis by RT-qPCR.

For RT-qPCR, total RNA was extracted from 2 to 5 × 10^4^ cells using an RNeasy 96 kit (Qiagen) and eluted in 100 μl of water. Real time PCR was performed on a Quantstudio 6 real-time PCR machine (Applied Biosystyem) in a 20-μl reaction consisting of 9 μl of total RNA, 10 μl of 2× master mix (TaqMan RNA-to-Ct 1-step kit; Applied Biosystems), 900 nM concentrations of each primer, and a 250 nM concentration of the probe. The primers and probes were synthesized by IDT, and the sequences are listed in Table S2B. Mouse β-actin (NM_007393) was used as a comparison reference (IDT assay ID Mm.PT.39a.22214843.g).

### RNA-Seq, ultra-low-input ChIP-Seq, and data processing.

For RNA-Seq, total RNA from 10^6^ control BV2 cells, Banf1 gene-edited cells, and Banf1-complemented cells were extracted using RNeasy minikit (Qiagen). The quality of the RNA samples was evaluated using Bioanalyzer 2100 (Agilent). Libraries were sequenced on Illumina HiSeq2500 platforms (Genome Technology Access Center, Washington University). The reads were aligned to the mm9 build using Bowtie2 to map clean reads to reference genes. RPKM values were determined using STAR aligner.

Ultralow Input ChIP-Seq was performed as described previously (50). Briefly, aliquots of 10^5^ cells were suspended in 20 μl of EZ nuclei isolation buffer (Sigma) and digested using 20 μl of MNase (NEB MNase buffer, 1 μl of MNase, and 3 mM dithiothreitol) for 5 min at 37°C, which was quenched by adding 4.4 μl of 100 mM EDTA and 4.4 μl of 1% Triton X-100 and 1% deoxycholate. Digested nucleosomes were diluted in complete ChIP buffer (20 mM Tris-HCl [pH 8.0], 2 mM EDTA, 150 mM NaCl, 0.1% Triton X-100, 5 mM sodium butyrate, and protease inhibitors [Roche]), precleared using Dynabeads (Invitrogen) for 1 h at 4°C, and subjected to immunoprecipitation overnight at 4°C (0.1 μg of H3K27ac; Abcam, ab4729). Bead chromatin complexes were washed sequentially using low-salt buffer (0.1% SDS, 1% Triton X-100, 2 mM EDTA, 20 mM Tris [pH 8.0], 150 mM NaCl) and high-salt buffer (0.1% SDS, 1% Triton X-100, 2 mM EDTA, 20 mM Tris [pH 8.0,] 500 mM NaCl) and then eluted with 1% SDS and 100 mM NaHCO_3_ for 1 h at 65°C. DNA was purified using Maxtract tubes (Qiagen) and precipitated overnight. For ATAC-Seq, aliquots of 5 × 10^4^ cells were processed according to the manufacturer’s instructions (Nextera DNA library preparation kit; Illumina).

Deep sequencing was performed on an Illumina HiSeq2500 1 × 50 (Genome Technology Access Center, Washington University). After demultiplexing, files were processed for alignment, peak calling, RPKM normalization, and visualization using NovaAlign, Macs2, DeepTool’s BamCoverage (51), and the UCSC Genome Browser (52), respectively. The R packages ChIPpeakAnno (53) and ggplot2 were used to assign H3K27 acetylation peaks to closest genes and plotting. Raw and processed data are available on the GEO database (GSE141386).

### Immunofluorescence microscopy.

For γH2AX staining, MEFs were seeded onto microscope coverslips and fixed for 25 min with 3.2% PFA in 1× phosphate-buffered saline (PBS). The cells then were washed extensively with IF wash buffer (1× PBS, 0.5% NP-40, 0.02% NaN_3_) and blocked with IF blocking buffer (IF wash buffer plus 10% FBS) for at least 30 min. Cells were incubated with mouse anti-pH2A.X antibody (Abcam, 26350; 1:1,000 in IF blocking buffer) for 1 h at 4°C. Cells were washed three times with IF washing buffer and then stained with secondary antibody (1:1,000; goat anti-mouse conjugated with Alexa Fluor 594; Invitrogen, catalog no. A11032) and Hoechst (1:5,000; BD Biosciences 33342) diluted in IF blocking buffer for 1 h at 4°C. Coverslips were rinsed extensively and then mounted using Prolong Gold mounting medium (Invitrogen, catalog no. P36930). Epifluorescence microscopy was performed on an Olympus fluorescence microscope (BX-53) using an UPlanS-Apo 60×/1.35 oil immersion lens with immersion oil from Millipore (104699). Images were obtained at room temperature using an ORCA-Flash4.0 LT digital camera (Hamamatsu, catalog no. C11440) and cellSens Dimension software. All images on the same channel were acquired using the same exposure time, and no digital gain was applied during image capture. Raw images were exported into Adobe Photoshop, and for any adjustments in image contrast or brightness, the levels function was applied. For focus quantitation, at least 100 cells were analyzed in triplicate.

For cytoplasmic double-stranded DNA staining, 8,000 BV2 cells in 250 μl of DMEM media were seeded into wells of a chamber slide (Nunc, Lab-Tek II) and cultured for 2 days. Cells were stained with MitoTracker Deep Red FM (Thermo Fisher, catalog no. M22426; 1 μM) in Opti-MEM at 37°C for 30 min, and then fixed with 4% PFA in PBS for 20 min at room temperature. Cells were washed with PBS and blocked with IFA buffer (0.1% saponin and 5% goat serum in PBS) for 10 min at room temperature. Cells then were stained with primary antibody (Santa Cruz, double-stranded DNA marker antibody [HYB331-01], sc-58749, diluted 1:100 in IFA buffer) at 4°C for 24 h. After washing with PBS and blocking with IFA buffer at room temperature for 10 min, the cells were incubated with a secondary antibody (Thermo Fisher, goat anti-mouse Alexa Fluor 488, catalog no. A11029; 1:250 in IFA) at room temperature for 1 h. After washing and staining with DAPI (4′,6′-diamidino-2-phenylindole; 1:500 in IFA buffer), cells were mounted with Prolong Gold mounting medium (Invitrogen, catalog no. P36930). Confocal microscopy was performed on a Zeiss LSM 880 confocal microscope using a Plan-Apochromat 63×/1.4 oil immersion lens (Zeiss). Images in each experiment were acquired at identical settings (detector gain of 590 and 600 for DAPI and Alexa Fluor 488, respectively; zoom, 1.5× for each). Images were analyzed with Volocity software (Quorum Technologies).

### Confocal microscopy imaging of mitochondria in live cells.

For live cell imaging, cells were grown in four-chamber glass-bottom dishes (D35C4-20-1.5-N; Cellvis). Cells were loaded with MitoTracker Green FM (200 nM; Thermo Fisher) together with Image-iT TMRM reagent (1:1,000; Thermo Fisher) in Live Cell Imaging Solution (Thermo Fisher, catalog no. A14291DJ) at 37°C for 30 min. Cells then were washed three times and imaged in live cell imaging solution at 37°C and 5% CO_2_. The cells were analyzed using Zeiss LSM 880 Airyscan confocal microscope (objective Plan-Apochromat 63×/1.4 oil; Zeiss) equipped with a Pecon stage-top incubator with controlled temperature and CO_2_. Images in each experiment were acquired at identical settings (Ex/Em 561/606, detection wavelength of 571 to 641, detector gain 646.2 for TMRM; Ex/Em 488/530, detection wavelength of 499 to 561, detector gain 837.0 for MitoTracker Green FM).

### Analysis of mitochondrial membrane potential by flow cytometry.

Cells were loaded with Image-iT TMRM reagent (1:1,000; catalog no. I34361; Thermo Fisher) in complete culture medium at 37°C for 30 min. The cells were then washed three times with PBS and resuspended in PBS. Analysis of the stained cells was carried out using Canto II flow cytometer (Becton Dickinson), and data were analyzed with FlowJo v10.6.1 software (Tree Star).

### Quantification of cytosolic DNA.

The method to isolate cytosolic DNA is modified from a published paper (54). Briefly, 10^6^ BV2 cells were placed in individual wells of a six-well plate and cultured at 37°C for 16 h. The cells were washed once with PBS and lysed by adding 100 μl of 1% NP-40. Lysates were scraped and gathered in a 1.5-ml tube and incubated on ice for 15 min. Lysates were centrifuged at 16,000 × *g* for 15 min at 4°C, and the supernatant and cell pellets were separated. The supernatant was subjected to DNA extraction with the DNeasy blood and tissue kit (Qiagen). The pellet was lysed with 180 μl of ALT buffer (DNeasy blood and tissue kit; Qiagen) and 20 μl of proteinase K, followed by incubation at 56°C for 10 min. DNA was extracted according to the manufacturer’s instructions. For both supernatants and pellets, DNA was eluted with 100 μl of AE (Tris-HCl-EDTA) elution buffer. The DNA was diluted 50× to load 7 μl per qPCR (SYBR green; KAPA HiFi HotStart). The abundance of a nuclear *18S* gene (forward primer, 5′-TAGAGGGACAAGTGGCGTTC-3′; reverse primer, 5′-CGCTGAGCCAGTCAGTGT-3′) and a mitochondrial gene, *Cox1* (mt-Cox1_F, TCGGAGCCCCAGATATAGCATT; mt-Cox1_R, CTGCTCCTGCTTCTACTATTGATG), was measured. The cytoplasmic DNA level was expressed as a ratio to those in the pellet.

### Statistical analysis.

Data from at least three independent experiments were pooled and subjected to a normality test prior to significance test. Nonnormal distribution data were log transformed. Significance was tested by one-way analysis of variance (ANOVA), with Dunnett’s posttest; two-way ANOVA, with Tukey’s posttest; or unpaired two-tailed *t* test. Data were analyzed using Prism version 8 (GraphPad). Statistical significance was assigned when *P* values were <0.05.
