# Supplementary material for: Barrier-to-Autointegration Factor 1 Protects against a Basal cGAS-STING Response
Source: mBio. 2020 Mar 10;11(2):e00136-20. doi: 10.1128/mBio.00136-20 (PMC7064753; doi:10.1128/mBio.00136-20)
Supplement: TABLE S2 [file mBio.00136-20-st002.docx]

**Table S2. sgRNA and qPCR primer sequences**

**A. sgRNA Sequences**

| Target Gene | Target Transcript | sgRNA Target Sequence |
| --- | --- | --- |
| Usp18 | NM_011909.2 | GCTCTCTCTTGCAGACAGAG |
| Stat1 | NM_001205313.1 | GTACGATGACAGTTTCCCCA |
| Aim2 | NM_001013779.2 | TGCCAGGAGCACACTCGACG |
| Aim2 | NM_001013779.2 | AGAGATATTTCATGCAACAG |
| Zbp1 | NM_021394.2 | TGAGCTATGACGGACAGACG |
| Zbp1 | NM_021394.2 | CAGGTGTTGAGCGATGACGG |
| Pqbp1 | NM_001252529.1 | AACACCTTGTACCAGCTCGG |
| Pqbp1 | NM_001252529.1 | AGAGCGCAACTACGACAAAG |
| Ddx41 | NM_134059.2 | ATGCTCAGGACATAACGCGG |
| Ddx41 | NM_134059.2 | ACGTACCCTATGTGCCGTTG |
| Pyhin1 | NM_175026.3 | GGGTCCAGAATACTGCTGGA |
| Pyhin1 | NM_175026.3 | TCCTCTAGCAACAATAGCCA |
| Irf1 | NM_008390.2 | GCTGTGTGGAGATGTTAGCC |
| Irf1 | NM_008390.2 | CTGTAGGTTATACAGATCAG |
| Irf3 | NM_016849.4 | GGCTGGACGAGAGCCGAACG |
| Irf3 | NM_016849.4 | CTGGCGGCCTCGGTAGAAGG |
| Irf8 | NM_008320.4 | AGTTTACCGAATTGTCCCCG |
| Irf8 | NM_008320.4 | TCGACAGCAGCATGTACCCG |
| Mb21d1(Cgas) | NM_173386.5 | GAGGCGCGGAAAGTCGTAAG |
| Mb21d1 (Cgas) | NM_173386.5 | AAATTCAAAAGAATTCCACG |
| Ifi204 | NM_008329.2 | CATGTTAGCAATCTGAATCG |
| Ifi204 | NM_008329.2 | ATGGCACAACATCAACTGCA |
| Tmem173(STING) | NM_028261.1 | AGTATGACCAGGCCAGCCCG |
| Tmem173(STING) | NM_028261.1 | CAGTAGTCCAAGTTCGTGCG |

**B. qPCR Primers**

| Gene | Primer (SYBR Green) or Taqman assay ID (IDT) |
| --- | --- |
| Actb | Mm.PT.39a.22214843.g |
| Rsad2 | Mm.PT.58.11280480 |
| Oas2 | Mm.PT.56a.7124473 |
| Ifit1 | Mm.PT.58.32674307 |
| Isg15 | Mm.PT.58.41476392.g |
| Ifnb1 | Mm.PT.58.30132453.g |
| Cxcl10 | Mm.PT.58.43575827 |
| Irf7 | Mm.PT.58.32394021.g |
| Bst2 | Mm.PT.58.6675636 |
| Actb | forward:TGTTACCAACTGGGACGACA |
|  | reverse:CCATCACAATGCCTGTGGTA |
| Rasd2 | forward:GTGAGCAATGGCAGCCTTAT |
|  | reverse:ACCACCTCCTCAGCTTTTGA |
| Ifit1 | forward:GTCAAGGCAGGTTTCTGAGG |
|  | reverse:AGGAACTGGACCTGCTCTGA |
